# UCSC Data Integrator and Variant Annotation Integrator

**DOI:** 10.1093/bioinformatics/btv766

**Published:** 2016-01-06

**Authors:** Angie S. Hinrichs, Brian J. Raney, Matthew L. Speir, Brooke Rhead, Jonathan Casper, Donna Karolchik, Robert M. Kuhn, Kate R. Rosenbloom, Ann S. Zweig, David Haussler, W. James Kent

**Affiliations:** ^1^Genomics Institute, University of California, Santa Cruz, CA, USA,; ^2^Computational Biology Graduate Group, University of California, Berkeley, CA, USA and; ^3^Howard Hughes Medical Institute, University of California, Santa Cruz, CA, USA

## Abstract

**Summary:** Two new tools on the UCSC Genome Browser web site provide improved ways of combining information from multiple datasets, optionally including the user's own custom track data and/or data from track hubs. The Data Integrator combines columns from multiple data tracks, showing all items from the first track along with overlapping items from the other tracks. The Variant Annotation Integrator is tailored to adding functional annotations to variant calls; it offers a more restricted set of underlying data tracks but adds predictions of each variant's consequences for any overlapping or nearby gene transcript. When available, it optionally adds additional annotations including effect prediction scores from dbNSFP for missense mutations, ENCODE regulatory summary tracks and conservation scores.

**Availability and implementation:** The web tools are freely available at http://genome.ucsc.edu/ and the underlying database is available for download at http://hgdownload.cse.ucsc.edu/. The software (written in C and Javascript) is available from https://genome-store.ucsc.edu/ and is freely available for academic and non-profit usage; commercial users must obtain a license.

**Contact**: angie@soe.ucsc.edu

**Supplementary information:** Supplementary data are available at *Bioinformatics* online.

## 1 Introduction

The UCSC Genome Browser database (Karolchik *et al.*, 2003; Speir *et al.*, 2016) contains a wealth of genomic datasets. One of its strengths is the suite of web tools at http://genome.ucsc.edu/ for visualizing and extracting data from the database in combination with the user's own custom track data as well as data provided via track hubs (Raney *et al.*, 2014). For over a decade, the Table Browser (Karolchik *et al.*, 2004) has provided the capability to extract textual data from any data track, with many options such as filtering by values, format conversion and sequence output. However, its abilities to combine data from multiple tracks are limited. It provides an intersection function that retains items in the selected track that overlap with items in a second track; however, the identities and attributes of items in the second track are not retained, so it is not possible to associate items in one track with items in another track. Over the years, many users of the UCSC Genome Browser have requested that capability, so we have developed a new tool, the Data Integrator (DI), to provide a flexible and open-ended query interface for combining data columns from multiple tracks.

One common request is to add annotations to a user's custom track of variant calls, for example the name of any gene that the variant intersects. Variant functional annotation is a well-studied problem (although by no means solved) for which many tools have been developed, such as the Ensembl Variant Effect Predictor (McLaren *et al.*, 2010), snpEff (Cingolani *et al.*, 2012) and ANNOVAR (Wang *et al.*, 2010). Inspired by those tools, we have added the Variant Annotation Integrator (VAI) with a focus on data tracks that may help to predict whether a given variant may modify a gene or regulatory region.

## 2 Data integrator

The Data Integrator is a single-page web application for building a query on Genome Browser tracks including user custom tracks and track hubs. It is reachable by the ‘Tools’ menu in the top navigation bar of the Genome Browser web site (http://genome.ucsc.edu). The ‘Help’ menu links to the Data Integrator User’s Guide (http://genome.ucsc.edu/goldenPath/help/hgIntegratorHelp.html).

The steps for building a query are as follows:

1. Select the genome and assembly version to use.
2. Select the genomic region(s) to annotate; the entire genome, the position range viewed in the Genome Browser, or a list of regions. The position range box accepts search terms such as gene symbols, cytobands, sequence accessions, or keywords.
3. Add data source(s) by selecting a track from menus in the ‘Add Data Source’ section and clicking the ‘Add’ button. Tracks can be dragged and dropped to change their order, or removed by clicking the ‘X’ icon. The track at the top of the list is the primary track; all of its items within the chosen region(s) will appear in the output. Items from the rest of the tracks are included only if they overlap an item from the primary track and are in the chosen region.
4. The output may be downloaded to a local file, optionally compressed with gzip, or may be viewed in the browser window. Click the ‘Choose fields…’ button to select or deselect data source columns to appear in the output.
5. Click the ‘Get output’ button to start the query.

The results of the query are returned as tab-separated text with selected columns of the primary data source followed by selected columns of additional data sources.

## 3 Variant annotation integrator

While the DI offers the entire set of tracks with none selected by default, the VAI requires variant calls as its input and requires a gene annotation track. A limited selection of additional tracks is offered. The benefit of this imposed query structure is that a more in-depth analysis of possible functional impacts of each variant can be performed.

Like the DI, the VAI is reachable from the Tools menu. Documentation appears following the configuration section.

Variant calls can be provided in Variant Call Format (VCF; Danecek *et al.*, 2011), Personal Genome SNP format (http://genome.ucsc.edu/FAQ/FAQformat.html#format10), or as a collection of dbSNP rs*NNNNN* identifiers.

The VAI predicts functional consequences based on the location of a variant within a gene transcript if applicable, using terms from the Sequence Ontology (SO; Eilbeck *et al.*, 2005) to facilitate downstream analysis and comparison of results with other variant analysis tools. For example, a single-base substitution in the coding region of a transcript is classified as synonymous_variant, missense_variant, stop_lost or stop_gained (See Supplementary Table S2 for the complete set of consequence SO terms used by the VAI.)

The gene annotation set should be chosen carefully, because small differences in transcript annotations can result in significant differences in predicted consequences (McCarthy *et al.*, 2014). The Genome Browser database includes a variety of gene annotation sets; experimentation in the VAI may help to choose the best one for a particular purpose.

The VAI offers additional data sources when they are available in the chosen assembly database; these may be added if desired. For identifying putative regulatory regions, two summary tracks from ENCODE (The ENCODE Project Consortium, 2012) are offered for hg19/GRCh37 and hg38/GRCh38: DNase Clusters and Transcription Factor ChIP-Seq peaks. For missense coding variants in hg19/GRCh37 and hg38/GRCh38, dbNSFP (Liu *et al.*, 2016) provides scores from several tools that predict likelihood of harm from an amino acid change. Variant identifiers from dbSNP (Wheeler *et al.*, 2007) are added if the variant coordinates match. Conservation scores and elements from phastCons (Siepel *et al.*, 2005) and scores from phyloP (Pollard *et al.*, 2010) can be added if available.

The user may add filters to reduce the volume of output, for example restricting the output to annotations with a particular consequence type or by overlap with common variants from dbSNP or conserved elements.

Output may be either an HTML-formatted table in the web browser window, or tab-separated text that can be viewed in the web browser window or downloaded as a file, optionally compressed by gzip. Columns are comparable to the output of the Variant Effect Predictor (McLaren *et al.*, 2010).

In order to make it clear to users that the VAI is only a research tool, and in no way should be used to inform medical decisions, a dialog pops up the first time a user gets output from the VAI, requiring a click-through agreement.

## 4 Conclusion

The DI and VAI offer two new, complementary ways to interactively mine data from the UCSC Genome Browser database, making a useful addition to the existing Table Browser.

Future plans for the DI include adding selection from related database tables where applicable, drag-reorder of output columns, filters on inputs and outputs and more options for configuring intersection of items. Future plans for the VAI include VCF output, HGVS notation (http://www.hgvs.org/mutnomen) and more annotation choices.

## Supplementary Material

Supplementary Data
