# Supplementary material for: UCSC Data Integrator and Variant Annotation Integrator
Source: Bioinformatics. 2016 Jan 6;32(9):1430–2. doi: 10.1093/bioinformatics/btv766 (PMC4848401; doi:10.1093/bioinformatics/btv766)
Supplement: Supplementary Data [file supp_32_9_1430__index.html]

UCSC Data Integrator and Variant Annotation Integrator — UCSC Data Integrator and Variant Annotation Integrator — UCSC Data Integrator and Variant Annotation Integrator — Supplementary Data 

# UCSC Data Integrator and Variant Annotation Integrator

## Supplementary Data

files

- Supplementary Data - docx file
