# Supplementary material for: UCSC Data Integrator and Variant Annotation Integrator
Source: Bioinformatics. 2016 Jan 6;32(9):1430–2. doi: 10.1093/bioinformatics/btv766 (PMC4848401; doi:10.1093/bioinformatics/btv766)
Supplement: Supplementary Data [file supp_btv766_supplementaryDataVaiDi.docx]

Databases and ontologies

UCSC Data Integrator and Variant Annotation Integrator

Angie S. Hinrichs^1,*^, Brian J. Raney^1^, Matthew L. Speir^1^, Brooke Rhead^2^, Jonathan Casper^1^, Donna Karolchik^1^, Robert M. Kuhn^1^, Kate R. Rosenbloom^1^, Ann S. Zweig^1^, David Haussler^1,3^  and W. James Kent^1^

^1^Genomics Institute, University of California, Santa Cruz, Santa Cruz, CA.

^2^Computational Biology Graduate Group, University of California, Berkeley, Berkeley, CA.

^3^Howard Hughes Medical Institute, University of California, Santa Cruz, Santa Cruz, CA.

**SUPPLEMENTARY DATA**

**LOCATION AND DOCUMENTATION**

The Data Integrator tool (DI) is at <http://genome.ucsc.edu/cgi-bin/hgIntegrator> . The most up-to-date documentation for the Data Integrator can be found at <http://genome.ucsc.edu/goldenPath/help/hgIntegratorHelp.html> .

The Variant Annotation Integrator tool (VAI) is at <http://genome.ucsc.edu/cgi-bin/hgVai> . Its documentation is on the same page, below the active section of the tool.

The UCSC Genome Browser database can be downloaded from <http://hgdownload.cse.ucsc.edu/> . In addition, the VAI uses several bigBed-formatted files derived from the full dbNSFP database that can be downloaded from <http://hgdownload.cse.ucsc.edu/gbdb/hg19/dbNsfp/> (hg19/GRCh37) and <http://hgdownload.cse.ucsc.edu/gbdb/hg38/dbNsfp/> (hg38/GRCh38). Other bigBed-formatted files containing condensed data for quick lookups, for example of dbSNP items and location, can be found at <http://hgdownload.cse.ucsc.edu/gbdb/hg19/vai/> .

**COMPARISON OF DATA INTEGRATOR AND TABLE BROWSER**

The Table Browser (TB) and Data Integrator (DI) share basic functionality such as retrieval of plain text representations of the database tables and binary files underlying Genome Browser tracks. However, each tool has capabilities that the other lacks, most notably:

- The TB allows the user to select a database table that does not have genomic coordinates (i.e. chromosome or scaffold, start, end), while the DI requires selection of one or more tracks with genomic coordinates.
- The TB offers several output formats such as FASTA sequence and GTF, and can create and load custom tracks, while the DI outputs only tab-separated text.
- The TB’s intersection function restricts the output to BED-compatible columns of the primary track, losing any columns that are not found in the BED format, and loses all information except genomic coordinates from the secondary track, while the DI allows selection of multiple tracks and can retain all columns of all tracks (or a selected subset).

The DI exists to perform a class of queries that the TB cannot: “output the columns of each item in track 1 with the columns of any overlapping item in track 2”. For example, “output each item in My Custom Track with the name, observed, exceptions and alleleNs columns from Common SNPs.” The DI will output the columns of the custom track plus the selected columns from Common SNPs (where there is an overlapping item in Common SNPs).

The TB can include or exclude items in track 1 based on whether they overlap items in track 2. It can also compute ranges covered by both tracks or by either track. However, it cannot include the name or other properties of items in track 2, because it distills all information in track 2 into a set of position ranges. For example, if one selects My Custom Track and then creates an intersection with Common SNPs, the TB will output items in the custom track that overlap with Common SNPs -- but it will not include any columns from Common SNPs.

Supplementary Table 1

Comparison of Selected Table Browser and Data Integrator Features

| **Feature** | **TB Support?** | **DI Support?** |
| --- | --- | --- |
| Output all fields of a track as text | Y | Y |
| Output selected fields of a track and related tables as text | Y | Y (Dec. 2015) |
| Output selected fields of more than one track | N | Y |
| Reorder columns in output | N | Anticipated 2016 |
| Filter items by value | Y | Anticipated 2016 |
| Select a track and keep only the items that overlap with items in another track | Y | Anticipated 2016 |
| Select a track and convert it to BED / custom track / hyperlinks | Y | N |
| Select a track and get genomic/cDNA sequence of items | Y | N |
| Select a multi-species alignment track and get MAF output | Y | N |
| Correlate wiggle tracks | Y | N |

In summary, the TB is here to stay; the DI fills in a gap in the TB’s functionality that requires a fundamentally different implementation.

**SELECTION OF SEQUENCE ONTOLOGY (SO) TERMS ASSIGNED BY VAI**

For the past several years we have informally coordinated Sequence Ontology terms with Ensembl, so that predicted consequences included in SNP details pages in the Genome Browser and assigned by VAI are generally consistent with Ensembl/VEP.

The authors of VEP, SnpEff and ANNOVAR have collaborated to produce a specification for including predicted consequences in the INFO column of VCF (<http://snpeff.sourceforge.net/VCFannotationformat_v1.0.pdf>). The specification includes guidance for reporting and ordering Sequence Ontology terms. The purpose of coordinating output formats is to support direct comparison of the output of different tools. When we add the ability to produce output in VCF format, we will follow that specification.

Supplementary Table 2

Sequence Ontology (SO) terms assigned by the Variant Annotation Integrator

| **SO term** | **Description** |
| --- | --- |
| [intergenic_variant](http://sequenceontology.org/browser/current_release/term/SO:0001628#_blank) | A sequence variant located in the intergenic region, between genes. |
| [upstream_gene_variant](http://sequenceontology.org/browser/current_release/term/SO:0001631#_blank) | A sequence variant located 5' of a gene. (VAI searches within 5,000 bases.) |
| [downstream_gene_variant](http://sequenceontology.org/browser/current_release/term/SO:0001632#_blank) | A sequence variant located 3' of a gene. (VAI searches within 5,000 bases.) |
| [5_prime_UTR_variant](http://sequenceontology.org/browser/current_release/term/SO:0001623#_blank) | A variant located in the 5' untranslated region (UTR) of a gene. |
| [3_prime_UTR_variant](http://sequenceontology.org/browser/current_release/term/SO:0001624#_blank) | A variant located in the 3' untranslated region (UTR) of a gene. |
| [synonymous_variant](http://sequenceontology.org/browser/current_release/term/SO:0001819#_blank) | A sequence variant where there is no resulting change to the encoded amino acid. |
| [missense_variant](http://sequenceontology.org/browser/current_release/term/SO:0001583#_blank) | A sequence variant, that changes one or more bases, resulting in a different amino acid sequence but where the length is preserved. |
| [inframe_insertion](http://sequenceontology.org/browser/current_release/term/SO:0001821#_blank) | An inframe non synonymous variant that inserts bases into in the coding sequence. |
| [inframe_deletion](http://sequenceontology.org/browser/current_release/term/SO:0001822#_blank) | An inframe non synonymous variant that deletes bases from the coding sequence. |
| [frameshift_variant](http://sequenceontology.org/browser/current_release/term/SO:0001589#_blank) | A sequence variant which causes a disruption of the translational reading frame, because the number of nucleotides inserted or deleted is not a multiple of three. |
| [initiator_codon_variant](http://sequenceontology.org/browser/current_release/term/SO:0001582#_blank) | A codon variant that changes at least one base of the first codon of a transcript. |
| [incomplete_terminal_codon_variant](http://sequenceontology.org/browser/current_release/term/SO:0001626#_blank) | A sequence variant where at least one base of the final codon of an incompletely annotated transcript is changed. |
| [stop_lost](http://sequenceontology.org/browser/current_release/term/SO:0001578#_blank) | A sequence variant where at least one base of the terminator codon (stop) is changed, resulting in an elongated transcript. |
| [stop_retained_variant](http://sequenceontology.org/browser/current_release/term/SO:0001567#_blank) | A sequence variant where at least one base in the terminator codon is changed, but the terminator remains. |
| [exon_loss](http://sequenceontology.org/browser/current_release/term/SO:0001572#_blank) | A sequence variant whereby an exon is lost from the transcript. (VAI assigns this term when an entire exon is deleted.) |
| [stop_gained](http://sequenceontology.org/browser/current_release/term/SO:0001587#_blank) | A sequence variant whereby at least one base of a codon is changed, resulting in a premature stop codon, leading to a shortened transcript. |
| [NMD_transcript_variant](http://sequenceontology.org/browser/current_release/term/SO:0001621#_blank) | A variant in a transcript that is already the target of nonsense-mediated decay (NMD), i.e. stop codon is not in last exon nor within 50 bases of the end of the second-to-last exon. |
| [intron_variant](http://sequenceontology.org/browser/current_release/term/SO:0001627#_blank) | A transcript variant occurring within an intron. |
| [splice_donor_variant](http://sequenceontology.org/browser/current_release/term/SO:0001575#_blank) | A splice variant that changes the 2-base region at the 5' end of an intron. |
| [splice_acceptor_variant](http://sequenceontology.org/browser/current_release/term/SO:0001574#_blank) | A splice variant that changes the 2-base region at the 3' end of an intron. |
| [splice_region_variant](http://sequenceontology.org/browser/current_release/term/SO:0001630#_blank) | A sequence variant in which a change has occurred within the region of the splice site, either within 1-3 bases of the exon or 3-8 bases of the intron. |
| [complex_transcript_variant](http://sequenceontology.org/browser/current_release/term/SO:0001577#_blank) | A transcript variant with a complex insertion or deletion (indel) that spans an exon/intron border or a coding sequence/UTR border. |
| non_coding_transcript_exon_variant | A sequence variant that changes exon sequence of a non-coding gene. |
| regulatory_region_variant | A sequence variant located within a regulatory region. |
| TF_binding_site_variant | A sequence variant located within a transcription factor binding site. |

**DESIGN AND IMPLEMENTATION OF DI AND VAI**

The Table Browser (TB) loads all rows in each search region into memory before it begins to produce output for that search region. (For genome-wide search, one search region is created for each chromosome or scaffold in the assembly.) This method simplifies the code and results in good overall performance. However, for large tables or complex queries, this can cause a long delay before the first output is produced, sometimes resulting in web timeouts. In order for the Table Browser to join tracks by position, it would have to hold the complete results of several independent queries in memory before producing output, which would increase the frequency of web timeouts.

To reduce memory usage and time before producing output, new data libraries were written with a streaming interface: downstream modules request data from data sources and other upstream modules one row at a time, so output is produced as soon as the first row has been processed together with overlapping rows from other data sources. Implementation details such as database and/or file accesses are encapsulated in the data source modules. Data sources are required to produce output sorted by genomic position, so joining items by their positions is a simple merging operation that requires no knowledge of the data passing through it except for genomic coordinates. These data libraries can be found as anno*.[ch] C code files in the kent/src/lib and kent/src/hg/lib directories of the UCSC Genome Browser source code (<https://github.com/ucscGenomeBrowser/kent>).

In order to maintain reasonable performance and efficiency in the implementation of data sources, the data source implementations often include some internal buffering – for example, the data source module for database tables (annoStreamDb.c) performs a sql query for up to 100000 rows and processes the sql result immediately in order to avoid sql connection problems, storing the returned rows in memory until all have been consumed by upstream modules. If upstream modules request still more rows, another query for up to 100000 rows is performed. The limit of 100000 rows avoids slowness caused by queries that return millions of rows (some tables have more than 100 million rows in total) and slowness caused by overly frequent sql operations that would be caused by a smaller limit. The use of internal buffering strategies adds some delay before first output is produced, while keeping overall performance reasonable.

In order to provide a more flexible and interactive user interface, the DI web user interface is implemented with new Javascript libraries (kent/src/hg/js/model/*, kent/src/hg/js/react/*, kent/src/hg/js/external/*) built upon the React JS (<https://facebook.github.io/react/>) and Immutable JS (<https://github.com/facebook/immutable-js>) libraries. The DI client-side Javascript code builds up a Javascript object containing all query parameters, and sends it as JSON to the server where the back-end C code (kent/src/hg/hgIntegrator/hgIntegrator.c) parses the JSON and configures data source modules, an integrator module (kent/src/lib/annoGrator.c) and an output module (kent/src/lib/annoFormatTab.c).
